# Integrated Chinese and Western Medicine for Acute Guillain-barré Syndrome Treatment

**DOI:** 10.1515/tnsci-2020-0007

**Published:** 2020-02-29

**Authors:** Liu Yang, Xiumin Zhao

**Affiliations:** 1 The Second Hospital of Hebei Medical University, Shijiazhuang, China; 2 Shanghai Dunlu Biomedical Technology Co., Ltd. Shanghai, China

**Keywords:** Guillain-Barré syndrome, traditional Chinese medicine, nerve conduction, activity of daily living, Sensory dysfunction

## Abstract

**Introduction:**

Guillain-Barré syndrome (GBS) is a worldwide demyelinating polyradiculopathy and polyneuropathy. Currently, there is no specific drug for GBS, and established treatment is generally based on immune-modulating treatment with plasma exchange or intravenous immunoglobulin in combination with supportive care. This study aimed to investigate the efficiency of integrated Chinese and Western medicine for acute GBS treatment.

**Methods:**

We enrolled 73 subjects, and randomly divided them into two groups: 35 cases in the traditional Chinese medicine (TCM) group, and 28 in the Control group. The Control group was treated with the common Western medicine for one month; and the TCM group was administrated with one month of common treatment combined with TCM medication.

**Results:**

Compared to the controls, TCM significantly enhanced the treatment efficiency in symptom expression, including the TCM syndrome score, the activity of daily living score, Hughes functional score and sensory dysfunction assessment. The total effective rate of the TCM group was 94.29%, significantly better than controls (78.59%). Moreover, TCM provide better improvement in motor nerve conduction functions (distal motor latency and motor conduction velocity) and sensory nerve conduction functions (sensory conduction velocity and sensory nerve action potential) in median nerve, ulnar nerve, and common fibular nerve.

**Conclusion:**

When combined with TCM administration, the GBS treatment could acquire better outcomes.

## 1 Introduction

Guillain-Barré syndrome (GBS) is a multifactorial and lethal inflammatory demyelinating polyradiculopathy and polyneuropathy, characterized by flaccid paralysis and acute demyelinating changes in the peripheral nervous system. It is characterized by symmetric limb paralysis, occasionally accompanied by limb paresthesia and autonomic dysfunction. About 20% GBS patients have a phenotype of respiratory paralysis [1, 2]. GBS can be caused by a range of infectious factors and acute traumas [3]. Diagnosis and management of GBS are complicated as its clinical presentation and disease course are heterogeneous, and no international clinical guidelines are currently available [4]. However, the pathology of GBS can be categorized into two types: acute inflammatory demyelinating polyneuropathy and acute motor axonal neuropathy. Almost all GBS patients would suffer from acute inflammatory demyelinating polyradiculoneuropathy, and this could be reflected by systematic analysis of the clinical and electrophysiological features.

Modern medical treatment majorly applies symptomatic and supportive therapies [5, 6]. IVIg and plasma exchange are equally effective treatments for GBS; and there are some other special treatments, such as immunoglobulin therapy, plasmapheresis therapy, hormone therapy, and cerebrospinal fluid filtration [4, 7, 8, 9]. Currently, there is no specific drug for GBS treatment, and classical treatments are generally based on etiological programs, combined with neurotrophic and anti-inflammatory approaches. Besides, intravenous injection of high-dose immunoglobulin was reported to yield beneficial outcomes, but it is expensive so far and may induce side effects. To date, this disease still needs effective supporting therapeutic strategies, for example traditional Chinese medicine (TCM) approaches. TCM has been reported about unique advantages in autoimmune neuritis, demyelinating and neurodegenerative diseases (such as Parkinson’s disease, Alzheimer’s disease, amyotrophic lateral sclerosis, autoimmune encephalomyelitis, etc.) [10, 11, 12, 13, 14, 15, 16, 17, 18]. In our pilot clinical experiment, we observed that when combined an empirical TCM formula with the current treatment of GBS, better outcomes could be acquired. This study aimed to investigate the efficiency of integrated Chinese and Western medicine for acute GBS treatment.

## 2 Material and methods

### 2.1 Patients

We collected the patients admitted to the Second Hospital of Hebei Medical University from July 2017 to December 2018.

The necessary diagnostic criteria for GBS were as follow [19, 20, 21, 22, 23]: (1) more than two limbs in paralysis; (2) the sputum reflex disappeared. And supportive diagnostic criteria included: (1) Clinical features: with a rapid symptom progress (reaching a peak) within 4 weeks, and a gradually recover after 4 weeks; (2) with symmetrical paralysis; (3) subjective sensory symptoms are more obvious compared to objective signs; (4) some patients exhibited cranial nerve symptoms (50% with bilateral facial paralysis, and often with tongue muscle and swallowing muscle paralysis); (5) with a slowdown of nerve conduction velocity.

The exclusion criteria for patients were as follow: (1) with a recent history of chemical abuse; (2) with abnormal porphyrin metabolism, which suggested an acute onset of porphyria; (3) with diphtheria infection or myocarditis; (4) with symptoms similar to lead-toxic neuritis and with strong evidences of plumbism; (5) confirmedly diagnosed acute poliomyelitis, botulism, toxic peripheral neuropathy (such as nitrofurantoin, dapsone, organophosphate poisoning, etc.).

Overall, we enrolled 73 subjects, and randomly divided them into two groups. One group (control) was treated with the common Western medicine; and the other (the TCM group) was administrated with the common Western medicine method combined with traditional Chinese medicine (TCM).

**Ethical approval**: The research related to human use has been complied with all the relevant national regulations, institutional policies and in accordance the tenets of the Helsinki Declaration, and has been approved by the authors’ institutional review board or equivalent committee.

**Informed consent**: Informed consent has been obtained from all individuals included in this study.

### 2.2 Therapy programs

The Western medicine treatment was as follow. (1) General treatments: in the acute phase, the patient was allowed to rest in bed and the limbs were placed in the functional position. Doctors carefully observed the vital signs, lung and heart function, kept the airway open, turned over the patient on time and discharge respiratory secretions. Those who have difficulty swallowing received nasogastric feeding and was paid attention to the balance of water and electrolytes. Symptomatic treatment was given when abnormalities were found in blood pressure, heart rhythm, defecation, and urination. (2) Intravenous infusion of 5% glucose or 0.9% normal saline plus vitamin B6 (0.2 g/250 ml) once a day; intramuscular injection of vitamin B12 (0.5 mg, once a day); and intravenous infusion of gamma globulin (0.4 g/kg/d) for 5 days.

The additional TCM treatment besides the Western medicine program in the TCM group was as follow. Each patient was given a TCM herbal formula for one month (one package per day). For each package, the herbs were boiled for half an hour, and 600 ml drug juice were acquired. The drug juice drug juice was taken twice per day: half in the morning and half in the evening. The herbal formula was composed by astragalus 60 g, raw licorice 12 g, atractylodes 10 g, phellodendron 10 g, coix seed 10 g, salvia miltiorrhiza 10 g, red peony root 10 g, poria cocos 12 g.

During the treatment process, safety indexes were continually observed, including vital signs, routine blood test indexes, routine urine indexes, acid-base balance, electrocardiogram, liver and kidney functions, adverse reactions or side effects due to medication.

Evaluation of the effect of combined therapy was performed at two time points (before and after one month of treatment), and different dimensions were assessed by according methods, like scale scores and electrophysiology tests.

### 2.3 TCM syndrome score

The scale was completed by the TCM physician of each patient. Scores are assigned based on the weight of each symptom in the TCM syndrome score system [24, 25, 26, 27]. A score of 4 or more was set for diagnosis, a score above 4 and below 8 indicated a mild syndrome, that above 8 and below 12 indicated a moderate type, and that greater than or equal to 12 implied a severe type. Thereby, the TCM syndrome score should be between 4 to 30.

### 2.4 Assessment of the activity of daily living

The activity of daily living was assessed by the Barthel indexes [28, 29, 30]. The items include the activity of eating, bathing, dressing, and so on (in all 10 items). This assessment has a full mark of 100, and a score below 60 means the patient has impaired self-care capability.

### 2.5 Limb function assessment

The limb function was assessed by Hughes functional score [31]. This score is between 0 and 6. A zero score refers to a healthy limb function and score 6 implied the patient dies. A higher score means a more severe function loss of limbs.

### 2.6 Sensory dysfunction assessment

The sensory dysfunction was determined by a questionnaire with 5 levels (0-4 score) [32]. A zero score refers to a normal sensory and the score 4 means feeling fading or disappearing beyond the shoulder. A higher score indicates a more severe situation of sensory function impairment.

### 2.7 Efficacy evaluation

The efficacy of each treatment was evaluated, and four outcomes were classified.

Cure: the muscle strength returned to normal, respiratory muscle paralysis and bulbar paralysis disappeared.

Significant efficacy: the muscle strength was improved for at least one grade (score), and the symptoms of respiratory muscle paralysis and bulbar paralysis disappeared within 10 days.

Improvement: muscle strength began recovering and no progression was found, respiratory muscle paralysis and bulbar paralysis symptoms were controlled.

Ineffectiveness: muscle strength did not recover, and paralysis was not improved.

### 2.8 Electrophysiological examination

We here applied the Dantec Cantata^TM^ electromyograph (Denmark, Danish) to examine nerve electrophysiological changes. For each patient, the skin temperature was maintained at 31°C or higher during examination. The sensory and motor conduction ability of median nerve, ulnar nerve and common fibular nerve were measured. The proximal end of each nerve was stimulated by skin electrode, and the compound action potential of the muscle was recorded by the skin electrode at the distal end. For motor nerve conduction, the distal motor latency (DML) and motor conduction velocity (MCV) were measured. And for conduction of sensory nerves, the antidromic technique method was performed to record the sensory conduction velocity (SCV) and sensory nerve action potentials (SNAPs). The stimulating electrodes were placed longitudinally over the nerve to avoid transversally oriented stimuli. A single stimulus was enough to obtain a sizeable action potential.

### 2.9 Statistical analysis

All the data were expressed as mean ± standard error. The homogeneity of variance was first tested. The paired t-test was used to compare between two time points. Student t-test was used to compare the mean values between two groups. The rank sum test was used for unequal variances. Fisher’s exact test was performed for efficiency rate comparison. A *P* value less than 0.05 was considered statistically significant.

## 3 Results

### 3.1 Clinical features of patients

There were 28 subjects in the control group, including 19 males and 9 females, aged 10 to 67 (37.8±14.0) years, among which 22 cases had prodromal symptoms within 2 weeks before onset. The mean time since onset was 6.3 days [1, 2, 3, 4, 5, 6, 7, 8, 9, 10, 11, 12, 13, 14]. There were 5 mild cases, 17 moderate, and 6 severe cases. There were 35 patients in the TCM group, including 24 males and 11 females, aged 12-63 (40.1±16.9) years. Before onset, 22 patients exhibited prodromal symptoms within 2 weeks. The TCM group had 6 mild cases, 20 moderate, and 9 severe cases. As table 1 shown, there were no statistically significant differences in age, gender, and disease period (P>0.05) in two groups, and the basal features were comparable.

**Table 1:** Clinical features of two groups of patients

Group	Cases	Age	Disease period
Control	28	40.143±16.896	6.75±3.82
TCM	35	37.771±13.988	6.31±4.46

### 3.2 TCM enhanced the treatment efficiency in symptom expression

**Figure 1:**
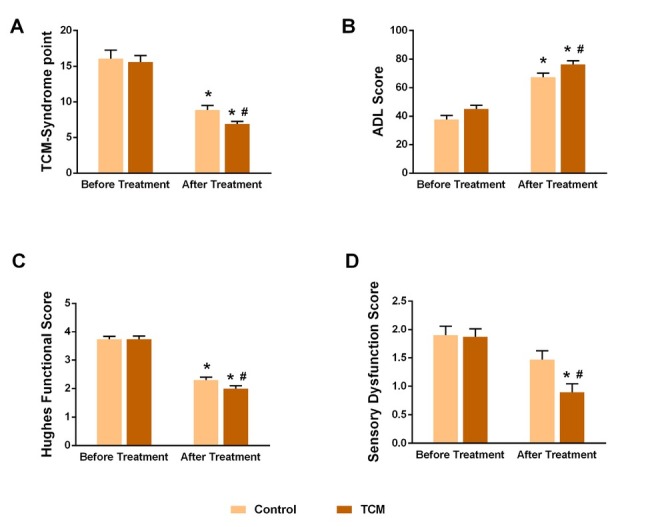
TCM enhanced the treatment efficiency in symptom expression. A. The TCM syndrome scores were significantly reduced after treatment in both groups of patients, we observed an even more dramatical improvement in the TCM group compared to controls. B. The activity of daily living (ADL) score was improved in both groups after treatment, patients in the TCM group exhibited higher ADL score compared to general treatment alone. C. TCM showed extra effects in Hughes functional score. D. The sensory dysfunction did not significantly decrease in paired comparison with the basal level for the control group, while it was significantly attenuated in the TCM group. *P < 0.05, paired t-test, after vs. before treatment, #P < 0.05, TCM vs. Control.

First, the TCM syndrome scores before and after treatment were analyzed between groups. No differences were found before treatment, and the scores were significantly reduced after treatment in both groups of patients (P < 0.05 for both groups, after treatment vs. before treatment). The TCM group showed an even more dramatical improvement when compared to controls (P < 0.05) (**Figure 1A**). Next, the ADL score was observed, and, consistently, both groups showed improved daily living activity after treatment (P < 0.05, vs. before treatment). When combined with TCM, the patients exhibited higher ADL score compared to general treatment alone (P < 0.05) (**Figure 1B**). Similar to ADL, the Hughes functional score suggested that although both therapies had benefited the limb function (P < 0.05, vs. before treatment), TCM showed extra effects in compared with control (P < 0.05) (**Figure 1C**). Further, the sensory dysfunction was evaluated for each patient. This parameter did not significantly decrease in paired comparison with the basal level for the control group, while it was significantly attenuated in the TCM group, referring to not only one month before (*P* < 0.05, vs. before treatment) but also the control group (*P* < 0.05) (**Figure 1D**). Moreover, the effective rate, as well as all subtypes, were listed in table 2. One month after treatment, two groups showed distinct effective rates. In the control group, there were 6 (21.43%) ineffective cases, 10 (35.71%) improved cases, 11 (39.29%) significantly effective cases, and 1 (3.57%) cured patient. The overall effective rate was 78.59%. In the TCM group, there accounted 2 (5.7%) ineffective, 8 (22.9%) improved, 20 (57.1%) significantly effective, and 5 (14.3%) cured cases. The total effective rate was 94.29%, significantly better than controls (Fisher’s Chi square = 6.7, *P* < 0.01).

**Table 2:** Efficiency of the general treatment and TCM combination

Group	Ineffectiveness	Improvement	Significant Efficacy	Cure	Overall Efficiency
Control	6 (21.4%)	10 (35.7%)	11 (39.3%)	1 (3.6%)	78.6%
TCM	2 (5.7%)	8 (22.9%)	20 (57.1%)	5 (14.3%)	94.3%**

**P < 0.01 TCM vs Control.

### 3.3 TCM improved nerve conduction functions

**Figure 2:**
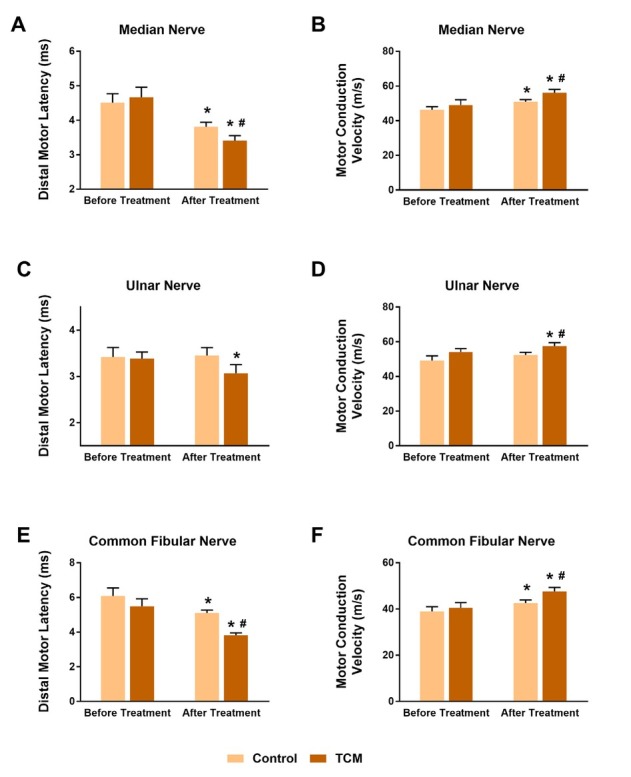
TCM improved motor nerve conduction functions in electrophysiological examination. A, B. The distal motor latency (DML) of median nerve was much shorter, and its motor conduction velocity (MCV) was higher in the TCM group. Both treatments had recovered the DML and MCV in comparison with the baseline. C, D. DML and MCV in ulnar nerve failed to recover in the control treatment, while they were significantly improved in the TCM group. Ulnar MCV in the TCM group was higher compared to the control. E, F. Both treatments contributed to the DML and MCV recovery in common fibular nerve; and TCM provided extra benefits compared to the control. *P < 0.05, paired t-test, after vs. before treatment, #P < 0.05, TCM vs. Control.

**Figure 3:**
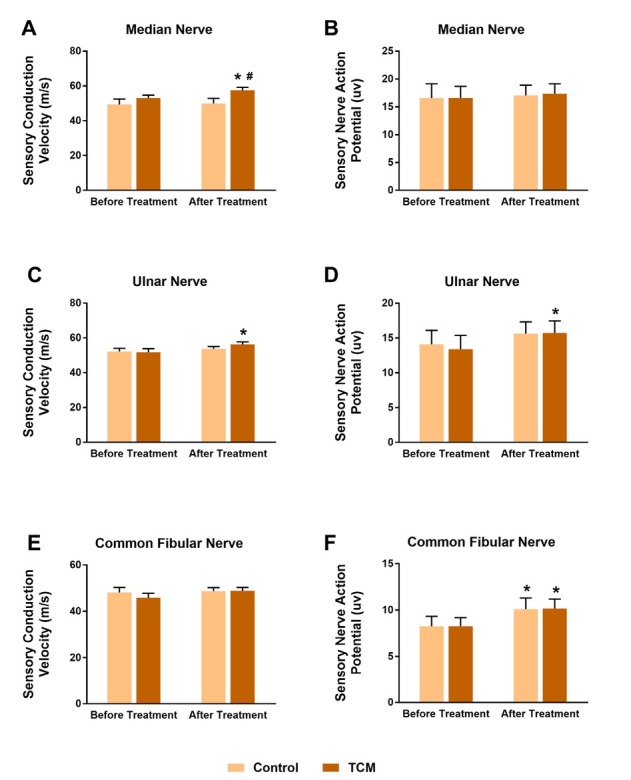
TCM improved sensory nerve conduction functions in electrophysiological examination. In the median nerve and ulnar nerve, sensory conduction velocity (SCV) and sensory nerve action potential (SNAP) values in the control patients after treatment were identical as before (A-D). Their SCV levels in the common fibular nerve were also unchanged (E), only sensory nerve action potential in the common fibular nerve was improved in the control group (F). TCM group showed significant increases in medium/ulnar-nerve SCV, and ulnar-nerve SNAP after treatment (A-D). The SCV value in the medium nerve was higher than the control group (A). The SNAP level in common fibular nerve kept unchanged in both groups under therapy administration (E). *P < 0.05, paired t-test, after vs. before treatment, #P < 0.05, TCM vs. Control.

Electrophysiological examination was performed as well at two time points, which aimed to reveal the nerve conduction mechanism underlying the symptom improvement. We majorly observed the sensory and motor conduction ability of median nerve, ulnar nerve and common fibular nerve. As **Figures 2** and **3** shown, each parameter had a similar comparable level between groups before treatment. In the aspect of motor nerve conduction, the TCM group exhibited an overwhelming advantage. The distal motor latency (DML) of median nerve was much shorter, and its motor conduction velocity (MCV) was higher after TCM combination treatment compared to the control (P < 0.05), when both treatments had recovered the DML and MCV in comparison with the baseline (P < 0.05) (**Figure 2A** and **2B**). The two parameters in ulnar nerve failed to recover in the control treatment (P > 0.05), while they were significantly improved in the TCM group (P < 0.05, vs. before treatment) (**Figure 2C** and **2D**). In addition, ulnar MCV in the TCM group was higher compared to the control (P < 0.05) (**Figure 2D**). As expected, both treatments contributed to the DML and MCV recovery in common fibular nerve (P < 0.05, vs. before treatment) (**Figure 2E** and **2F**); and TCM provided extra benefits compared to the control (P < 0.05) (**Figure 2E** and **2F**). Sensory nerve conduction is another crucial indication of neurologic function and limb recovery. Sensory parameters were recorded, especially sensory conduction velocity (SCV) and sensory nerve action potential (SNAP). In the median nerve and ulnar nerve, SCV and SNAP values in the control patients after treatment were identical as before (P > 0.05) (**Figure 3A-D**); besides, their SCV levels in the common fibular nerve were also unchanged (P > 0.05) (**Figure 3E**); only sensory nerve action potential in the common fibular nerve was improved in the control group (P < 0.05, vs. before treatment) (**Figure 3F**). On the other hand, the TCM group showed significant increases in medium/ ulnar-nerve SCV, and ulnar-nerve SNAP after treatment (P < 0.05, vs. before treatment) (**Figure 3A-D**). Moreover, the SCV value in the medium nerve was higher than the control group (P < 0.05) (**Figure 3A**). The SNAP level in common fibular nerve kept unchanged in both groups under therapy administration (P > 0.05) (**Figure 3E**). Collectively, combination with TCM strongly enhanced the motor and sensory nerve conduction functions compared to general treatment alone.

## 4 Discussion

GBS is commonly known to include acute infectious demyelinating polyneuropathy and often shows a rapidly development of multiple peripheral neuropathy. It is also a sort of autoimmune disease and a rare but severe neurologic complication after trauma. At the beginning of GBS, inflammatory cells may disintegrate Schwann cells and phagocytose the myelin sheath, causing segmental demyelination, and occasionally even secondary axonal degeneration [33, 34]. When controlled timely, Schwann cells may begin to proliferate after two weeks of acute demyelination, followed by remyelination and inflammation extinction. As mentioned above, there is no specific drug for GBS treatment, and classical treatments urgently need more supporting therapeutic strategies, e.g. TCM approaches.

Very few researchers have probed to combine TCM medication with current classical GBS treatments. Only one published study suggested that tripterygium polyglycoside, a Chinese herbal medicine, hastened GBS recovery more rapidly than corticosteroids [35]. However, the etiology of GBS is so complicated that any effect of a single component or herb could be extremely limited. The clinical manifestations of GBS are highly similar to the category of flaccidity disease in TCM theory, which might be caused in part by lack of Qi and Yin, as well as “evil heat”, and could be treated by the tonifying-Qi herbs (e.g. astragalus, licorice and Poria cocos) and the removing-damp-heat herbs (e.g. atractylodes, cork, coix seed, radix paeoniae and salvia miltiorrhiza). Here we first proved that compound TCM significantly enhanced the efficiency of common Western medicine in GBS treatment, not only in TCM-symptom scores but also ADL, limb function and sensory function.

In the TCM theory, a lack of Qi is associated with impaired limb function and activities of daily living [36, 37, 38, 39], and the damp-heat evil (a pathogenic factor formed by combination of heat and dampness evils) can cause inflammatory/pain reaction combined with a damage in capacity for action [40, 41, 42]. In the acute phase of GBS, there are frequently symptoms including extensive peripheral nerve demyelination and inflammatory response, motor nerve damage, and even vegetative nerve damage. The performance of these dysfunctions is in consistency with damp-heat syndromes, and theoretically (in TCM), the patients are under the condition of Qi lacking. Given the TCM syndrome differentiation on GBS, our formula mainly applied two strategies: tonifying-Qi and removing-damp-heat. Astragalus and Poria cocos are widely used and low-cost tonifying-Qi herbs in China [39, 43], and licorice is a tonifying-Qi drug with a detoxifcation capability (towards the damp-heat evil)[44, 45]; besides, Poria cocos can also fight the damp evil and accelerate the recovery of limb function [46, 47]; atractylodes and semen coicis are commonly used removing-damp-heat herbs [48, 49, 50], and the combination of salvia, red peony root and phellodendron can remove heat evil in blood and tissues [51, 52, 53]. Together, this TCM formula aimed to cure GBS according to the most frequent performance in the TCM syndrome differentiation when combined with routine medicine treatments.

For those patients divided in the TCM group, we chose an empirical formula to aid the classical treatment. Our formula was expected to exert an additional therapeutic role in GBS recovery theoretically. Wherein, astragalus polysaccharide could promote angiogenesis and fasten the tissue repair [54]. Licorice root shares a similar function to astragalus. Also, licorice has an anti-inflammatory and estrogen-like activity, which helps to attenuate the GBS symptoms [45, 55]. Salvia miltiorrhiza and radix paeoniae are effective in promoting limb function recovery through improving blood supply [56, 57]. Notably, it was reported that that severe fatigue after Guillain-Barré syndrome is related to more pronounced axonal loss [58]. In our TCM prescription, a group of tonifying-Qi herbs had a clear anti-fatigue role [59, 60, 61, 62]. This could be a mechanism of the recovery promoting effect shown in the TCM group.

Electrophysiological examination provides an auxiliary diagnosis for GBS, especially for classifying GBS into subgroups. For those GBS patients with respiratory paralysis, phrenic nerve electrophysiological abnormalities could be applied as important indicators and predictors [63, 64]. Electrodiagnosis was suggested to apply based on various reported phenomenon, such as reversible conduction failure, abnormal distal compound action potentials, etc. [20, 65, 66]. Under demyelination, GBS may lead a decreased motor conduction velocity and prolonged F wave latency in more than 70% of patients [67, 68]. And successful treatment of GBS was known to be accompanied with decreased DMLs, according to Hosokawa et al [66]. In the present study, we observed that GBS treatment decreased the DML and elevated the MCV. This was consistent with known studies. Overall, the changes of SNAP in GBS patients were not significant based on our study, but TCM could intensify the SNAP in ulnar and common fibular nerves. This suggest that there may exist different mechanisms about SNAP changes underlying the onset of GBS and the TCM treatment. Some published studies have also implied abnormal SNAPs (like absent SNAP) in GBS [69]. There are still controversies about whether GBS induces decreased SNAPs so far [65], and more investigations are merited. Finally, a proportion of GBS patients are electrophysiologically unclassified because the underlying pathophysiological mechanisms and lesion distributions are not well defined [66]. Our results may provide some commonalities in electrophysiological changes of GBS, in particular in impaired motor conduction.

## 5 Conclusion

In conclusion, when combined with TCM administration, the GBS treatment could acquire improved outcomes, including better performance in TCM-symptom score, ADL score, Hughes functional score, sensory dysfunction score, motor nerve conduction and sensory nerve conduction.

## Abbreviations

DML: distal motor latency
GBS: Guillain-Barré syndrome
MCV: motor conduction velocity
SCV: sensory conduction velocity
SNAPs: sensory nerve action potentials
